# Stochastic processes drive the soil fungal communities in a developing mid-channel bar

**DOI:** 10.3389/fmicb.2023.1104297

**Published:** 2023-02-06

**Authors:** Fei Ye, Yiguo Hong, Xuemei Yi, Zhaohong Sun, Jiapeng Wu, Yu Wang

**Affiliations:** ^1^Institute of Environmental Research at Greater Bay Area, Key Laboratory for Water Quality and Conservation of the Pearl River Delta, Ministry of Education, Guangzhou University, Guangzhou, China; ^2^Chongqing Institute of Green and Intelligent Technology, Chinese Academy of Sciences, Chongqing, China

**Keywords:** soil fungi, rhizosphere, community assembly, stochastic processes, water flooding, mid-channel bar

## Abstract

Intricate associations between rhizosphere microbial communities and plants play a critical role in developing and maintaining of soil ecological functioning. Therefore, understanding the assembly patterns of rhizosphere microbes in different plants and their responses to environmental changes is of great ecological implications for dynamic habitats. In this study, a developing mid-channel bar was employed in the Yangtze River to explore the assembly processes of rhizosphere fungal communities among various plant species using high-throughput sequencing-based null model analysis. The results showed a rare significant variation in the composition and alpha diversity of the rhizosphere fungal community among various plant species. Additionally, the soil properties were found to be the primary drivers instead of plant species types. The null model analysis revealed that the rhizosphere fungal communities were primarily driven by stochastic processes (i.e., undominated processes of ecological drift), and the predominance varied with various plant species. Moreover, the assembly processes of rhizosphere fungal communities were significantly related to the changes in soil properties (i.e., soil total carbon, total nitrogen, organic matter, and pH). The co-occurrence network analysis revealed that many keystone species belonged to unclassified fungi. Notably, five network hubs were almost unaffected by the measured soil properties and aboveground plant traits, indicating the effect of stochastic processes on the rhizosphere fungal community assembly. Overall, these results will provide insights into the underlying mechanisms of fungal community assembly in the rhizosphere soils, which are significant for maintaining the functional stability of a developing ecosystem.

## Introduction

Plant-microbe associations are essential for plants to successfully colonize and subsequently thrive in natural ecosystems, especially in developing habitats, which is crucial for ecosystem stability and functioning (Zhalnina et al., 2018; Hong et al., 2022; Ye et al., 2022). For instance, plants can regulate the associations between plants and soil microorganisms to resist environmental stress by producing various metabolites (Zhalnina et al., 2018; Hong et al., 2022). These associations are reinforced in the rhizosphere, where microorganisms play a predominant role in shaping plant community and productivity by enhancing nutrient acquisition, resource utilization, and stress resistance (Cantarel et al., 2015; Wei et al., 2020). Notably, soil fungi consisting of essential components of rhizosphere microbial diversity play a major role in driving critical biogeochemical cycles in rhizosphere soils, such as organic matter (OM) decomposition (Brundrett and Tedersoo, 2018). As such, the composition of rhizosphere fungal communities has a significant effect on complex associations among plants, microbes, and soil properties (Zhalnina et al., 2018).

Determining the effects of stochastic processes (i.e., homogenizing dispersal, dispersal limitation, and undominated processes) and deterministic processes (i.e., homogeneous and variable selection) on the assembly processes of the microbial community can reveal the structure and function of microbial community (Stegen et al., 2012; Powell et al., 2015; Shi et al., 2020). So far, the assembly processes of soil microbial communities have been extensively explored in various ecosystems, including grassland (Ji et al., 2020), orchard (Zheng et al., 2021), cropland (Jiao and Lu, 2020b), forests (Qin et al., 2022). Additionally, many studies have reported the responses of these assembly processes to different circumstances, such as hydrocarbon contamination (Potts et al., 2022), land cover alternation (Zhao et al., 2022), long-term fertilization (Shi et al., 2020), wildfire (Qin et al., 2022). However, the assembly processes of the microbial communities in a developing habitat have not been fully elucidated yet.

The predominance of stochastic and deterministic processes in microbial community assembly is regulated by several biotic and abiotic factors (Zheng et al., 2021; Shi et al., 2022; Zhao et al., 2022). Previous studies have reported that the relative contributions of those assembly processes were driven by the changes in soil pH (Jiao and Lu, 2020b), OM (Dini-Andreote et al., 2015; Zheng et al., 2021), inorganic nitrogen (Wan et al., 2021; Zhao et al., 2022), and available sulfur (Jiao and Lu, 2020a). Additionally, plant growth period (Zhao et al., 2022), foliar insect (Shi et al., 2022), and microbial substrate preferences (Zhalnina et al., 2018) could alter the assembly processes by governing soil microbial communities. However, the effect of plant species on the assembly processes of the rhizosphere microbial community, especially the fungal community, is still unclear. This understanding of the functional establishment and maintenance of a developing ecosystem is of great significance.

The present study focused on the mid-channel bar ecosystems existing in the form of islands surrounded by river water (Wen et al., 2020), which are vital for river channel stability and provisioning the ecosystem biodiversity and habitat (Tabacchi et al., 2009; Lou et al., 2018; Tonkin et al., 2018). The mid-channel bars are under long-term perturbation of water level fluctuation and sedimentation triggered by natural river regimes, inducing their geomorphic characteristics in the developing state (Hooke and Yorke, 2011). This study aimed to reveal the assembly processes and underlining driving factors of fungal communities in rhizosphere soils of different plant species in a mid-channel bar using high-throughput sequencing-based null model analysis. In our previous study, no significant difference was observed in the rhizosphere bacterial communities among different plant species (Ye et al., 2022). It was hypothesized that (i) plant species have no significant effect on the structures of rhizosphere fungal communities in the mid-channel bar; (ii) stochastic processes dominate the assembly of rhizosphere fungal communities.

## Materials and methods

### Description of the study site

**FIGURE 1:**
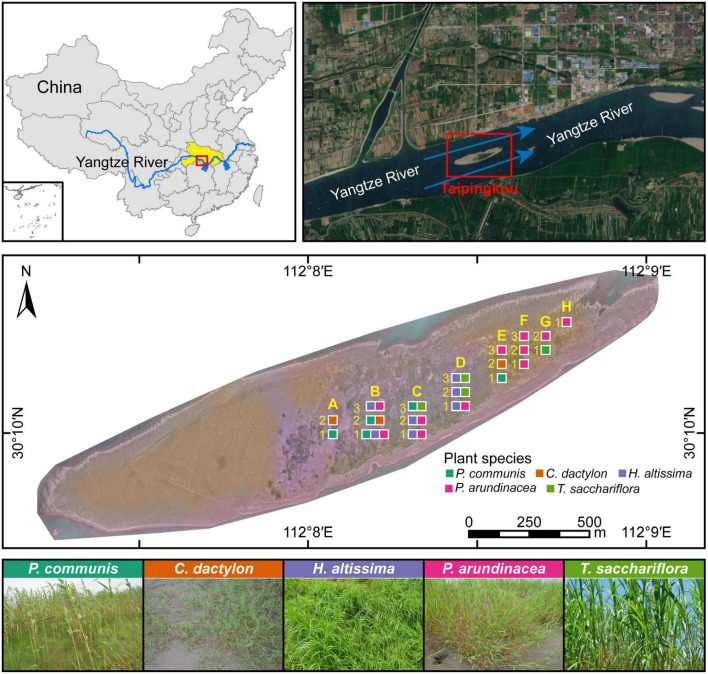
Location of the study site, Taipingkou mid-channel bar, in the upper Jingjiang section of the Yangtze River. Blue arrows indicate the flow direction of the Yangtze River. Yellow capital letters and numbers indicate the positions of the sampling quadrats in the transverse and longitudinal directions, respectively. Squares with different color represent the type of dominant plant species exists in each quadrat.

In this study, a mid-channel bar (Taipingkou: 30°30′ N, 112°13′ E) located at the middle reaches of the Yangtze River was selected as to investigate the assembly processes of rhizosphere fungal communities (Figure 1). The mid-channel bar formed in the last 50 years is regarded as a newborn, which has the tendency to move downstream continuously due to constant flow erosion and sediment deposition at the head and tail, respectively (Wang et al., 2015; Wen et al., 2020). As a sandy bar in the middle of the river channel (Wang et al., 2015), Taipingkou is relatively less affected by anthropogenic pollution inputs. However, it is more influenced by the changes in the hydrological regime and sediment deposition caused by the upstream dam operation. The detailed climatic conditions of the study area can be found in the previous article (Ye et al., 2022).

### Quadrat delineation and plant survey

Quadrat delineation and rhizosphere soil collection were performed during a survey on 26 May 2019. Along the mid-channel bar, 5 × 5 m quadrats were setup, which were uniformly distributed in 50 × 50 m sampling grids with a 25-m interval in between (Supplementary Figure 1). A total of 20 quadrats from three elevation-based transects were selected for plant survey and rhizosphere soil collection, including eight quadrats for transect one, seven quadrats for transect two, and five quadrats for transect three (Figure 1). In each sampling quadrat, the above-ground traits, including the number and coverage of each plant were recorded. The species-specific height of each plant was obtained by measuring the five randomly selected individuals. The importance value of each plant was calculated based on its relative height, frequency, and coverage (Ye et al., 2022). The dominant plants in each sampling quadrat were identified according to their importance values.

Five dominant plant species (*Phragmites communis*, *Cynodon dactylon*, *Hemarthria altissima*, *Phalaris arundinacea*, and *Triarrhena sacchariflora*) were confirmed across the sampling quadrats (Figure 1 and Supplementary Figure 2). *P. communis*, *C. dactylon*, *H. altissima*, *P. arundinacea*, and *T. sacchariflora* was dominant in four, four, seven, twelve and three quadrats, respectively. The values of above-ground traits (i.e., height, richness, coverage, and importance value) of these five plant species are presented in Supplementary Figure 2. Later, the importance value was calculated as the average of the sum of relative height, frequency, and coverage (Ye et al., 2022).

### Rhizosphere soil sampling

The rhizosphere soil samples were collected according to a previously reported method (Ye et al., 2021). Briefly, five plants of the same species were randomly selected and carefully removed from each quadrat. Soils loosely attached to the roots were gently shaken off. Soils tightly adhered to the roots were regarded as rhizosphere soils, and extracted using saline phosphate buffer. The rhizosphere soils of the same plant species in each quadrat were thoroughly mixed to form a composite sample.

The resulting 30 composite rhizosphere soils were subsequently divided into two parts and either stored at −20°C for DNA extraction or at 4°C for physicochemical analysis. The physicochemical property values of rhizosphere soils are presented in Supplementary Figure 2.

### DNA extraction, sequencing, and data processing

About 0.25 g of the rhizosphere soil from each composition sample was used to extract the DNA following the protocol of the DNeasy PowerSoil Kit (Qiagen, Hilden, Germany). The quality of the DNA was checked by 1% (w/v) agarose gel electrophoresis, and the concentration of the DNA was determined by a NanoDrop Lite Spectrophotometer (Thermo Scientific, Waltham, USA). The extracted DNA was stored in a −80°C refrigerator for subsequent analysis.

The fungal ITS gene was amplified by the polymerase chain reaction (PCR) using primers ITS1F (5′-CTTGGTCATTTAGAGGAAGTAA-3′)/ITS2R (5′-GCTGCGTTCTTCATCGATGC-3′) (Adams et al., 2013). The PCR products were purified using an AxyPrep DNA Gel Extraction Kit (Axygen, Union City, USA). The purified amplicons were then pooled in equimolar and paired-end sequenced on an Illumina MiSeq PE300 platform (Majorbio Bio-Pharm Technology Co., Ltd., Shanghai, China).^1^ The raw sequence data were demultiplexed and quality-filtered using Trimmomatic (v. 0.30) (Bolger et al., 2014). The operational taxonomic units (OTUs) were identified at 97% sequence similarity using USEARCH (v. 7.0).^2^ The fungal taxonomy was analyzed *via* the ribosomal database project (RDP) using the UNITE 8.0 database.^3^ The sequences in each sample were filtered to an equal sequencing depth based on the fewest sequence reads to compare the samples. Alpha diversity indices, such as Sobs, Shannon diversity, Heip’ evenness, and Phylogenetic diversity, were calculated using Mothur (v. 1.30.1). PCR, sequencing, and data processing were conducted according to a previously reported method (Ye et al., 2021).

### Null model analysis

A null model approach based on two indices of β-nearest taxon index (βNTI) and raup-crick index (RCI) was introduced to determine the assembly processes of soil fungal communities in different plant species. These two indices were calculated by the “picante” package in R v. 4.0.5 (Wan et al., 2021; Zhao et al., 2022). A βNTI value > 2 or < −2 indicates the dominance of deterministic processes (i.e., variable selection and homogeneous selection) on the assembly of a given microbial community. Similarly, the βNTI value < −2 suggests the effect of homogeneous selection and > 2 variable selection (Stegen et al., 2015). The value of βNTI ranging from −2 to 2 indicates the primary effect of stochastic processes (i.e., dispersal limitation, homogenizing dispersal, and undominated processes). The dispersal limitation is often regarded as the main driver of the community assembly with an RCI value larger than 0.95, while the homogenizing dispersal is identified as the primary contributor with an RCI value smaller than −0.95. Additionally, the RCI value ranging from −0.95 to 0.95 indicates the effect of undominated processes (Stegen et al., 2015).

### Co-occurrence network analysis

The potential associations between rhizosphere fungal communities were explored by the co-occurrence network analysis. The network was constructed based on the random matrix theory (RMT) using the molecular ecological network analysis pipeline (MENA)^4^ at an OTU level. Only OTUs present in 10 samples (one–third of the total samples) were retained based on the rarefied fungal OTU abundance table. Subsequently, the relative abundance data of OTUs were uploaded into MENA to conduct network analyses with the default settings and recommended cutoff threshold of 0.81 (Zhou et al., 2011; Deng et al., 2012; Ye et al., 2021). Visualization and layout of the network were performed based on the Fruchterman-Reingold algorithm using Gephi 0.9.2 (Bastian et al., 2009). The topological properties of the constructed network were calculated to describe the associations between the fungal taxa (Ma et al., 2016). Moreover, 1,000 Erdös–Réyni random networks were generated on the pipeline with an identical number of nodes and edges as the constructed network. The potential ecological role of each node in the network was confirmed based on the threshold of within-module connectivity (*Zi*) and among-module connectivity (*Pi*) at 2.5 and 0.62, respectively (Deng et al., 2012; Wang et al., 2020; Ye et al., 2021). Furthermore, the nodes highly connected to other nodes in a module were defined as the module hubs with *Zi* > 0.25 and *Pi* < 0.62; the nodes connected to different modules were defined as the connectors with *Zi* < 0.25 and *Pi* > 0.62; the nodes acting as a module hub and a connector were defined as the network hubs with *Zi* > 0.25 and *Pi* > 0.62 (Zhang et al., 2021).

### Statistical analyses

The statistical significances of dominant genera (relative abundance > 1% of the total sequences) and alpha diversity indices (i.e., Sobs, Shannon diversity, Heip’s evenness, and Phylogenetic diversity) of rhizosphere fungal communities among different plant species were determined by one-way analysis of variance (ANOVA) based on the least significant difference (LSD) using SPSS Statistics 20.0 (IBM, USA). The community distribution of rhizosphere fungi was assessed by the principal co-ordinates analysis (PCoA) based on the Bray–Curtis distance using CANOCO 5 software (Ter Braak and Šmilauer, 2012). The significant differences between rhizosphere fungal communities among different plant species were determined by analysis of similarity (ANOSIM) using R v. 3.5.1 with “vegan” package. The relationship between alpha diversity indices and environmental factors and the aboveground plant traits were determined by Spearman’s correlations using SPSS Statistics 20.0 (IBM, USA). The relationships between the fungal community composition and environmental factors, aboveground plant traits were explored by R v. 4.0.5 with “ggcor” package. The Pearson correlation coefficient was conducted in Origin 2022 (OriginLab, USA).

### Accession numbers

The raw sequencing data have been deposited in the National Center for Biotechnology Information (NCBI) Sequence Read Archive (SRA)^5^ under the accession number PRJNA903773.

## Results

### Composition, diversity, and impact factors of rhizosphere fungal community

**FIGURE 2:**
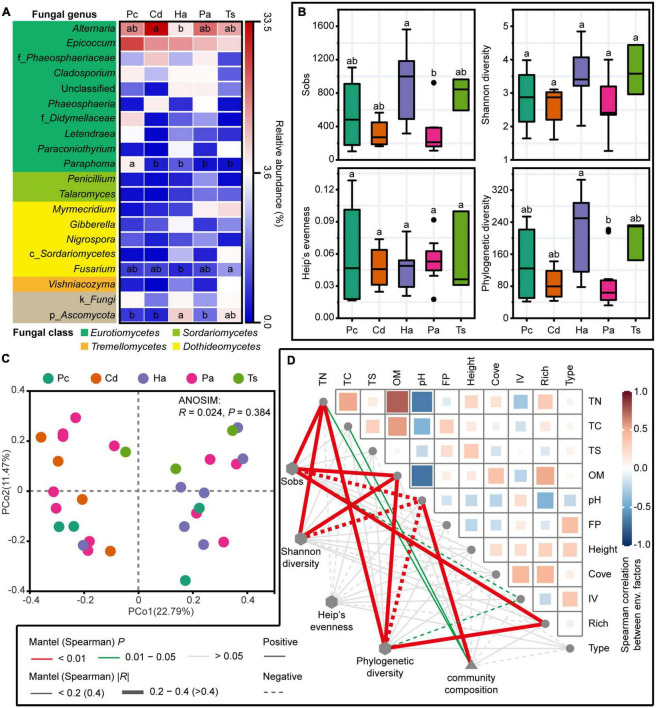
Fungal communities in rhizosphere soils. **(A)** The dominant fungal genera (sequences accounting for > 1% of the total sequences of all samples) and **(B)** alpha diversity indices of fungal communities in rhizosphere soils across different plant species. Different lowercase letters indicate significant differences among different plant species at 0.05 level of significance based on one-way analysis of variance (ANOVA) with least significant difference (LSD) multiple comparisons. Pc, *P. communis*; Cd, *C. dactylon*; Pa, *P. arundinacea*; Ha, *H. altissima*; Ts, *T. sacchariflora*. **(C)** Principal co-ordinate analysis (PCoA) of rhizosphere fungal communities at operational taxonomic unit (OTU) level based on Bray–Curtis distance. Analysis of similarity (ANOSIM) of rhizosphere fungal communities among different plant species based on Bray–Curtis distance with 999 permutations. **(D)** Correlations of fungal community diversity and composition with soil environmental factors and aboveground plant traits. Spearman’s correlations were performed between alpha diversity indices, soil environmental factors and plant traits. Mantel tests were executed between fungal community composition and soil environmental factors and plant traits. FP, flooding probability; Cove, coverage of plant species; IV, importance value; Rich, richness of plant species; Type, type of plant species.

A total of 1,934,139 high-quality sequences were acquired from 30 rhizosphere soils. These sequences were resampled to an even sequence depth of 41,749 across all samples based on the lowest sequence of samples. Subsequently, 4,615 OTUs were identified based on the rarefied sequences with 97% similarity. Among the dominant fungal genera (sequences accounting for more than 1% of the total sequences of all samples), *Alternaria* and *Epicoccum* were the two most abundant taxa, accounting for 18.1 and 15.2% of the total sequences, respectively (Figure 2A). The relative abundance of *Alternaria* in rhizosphere of *C. dactylon* was significantly higher than that in rhizosphere of *H. altissima* (one-way ANOVA, *P* < 0.05), and not significantly different from that of *P. communis*, *P. arundinacea*, and *T. sacchariflora* (one-way ANOVA, *P* > 0.05; Figure 2A). The relative abundance of *Epicoccum* in rhizosphere showed no significant difference among different plant species (one-way ANOVA, *P* > 0.05; Figure 2A).

The alpha diversity indices (i.e., Sobs, Shannon diversity, Heip’s evenness, and Phylogenetic diversity) of rhizosphere fungal communities did not exhibit significant differences among different plant species (one-way ANOVA, *P* > 0.05; Figure 2B). Exceptionally, significant differences were found between the Sobs and Phylogenetic diversity of *H. altissima* and *P. arundinacea* with higher values in the rhizosphere of *H. altissima* (one-way ANOVA, *P* < 0.05; Figure 2B). The Sobs and Phylogenetic diversity indices of rhizosphere fungal communities showed significant positive correlations with rhizosphere soil variables of total nitrogen (TN), OM and the aboveground plant trait of richness, but were negatively correlated with soil pH and the aboveground plant trait of importance value (Spearman’s correlations, *P* < 0.05; Figure 2D).

The PCoA showed an unorganized distribution of rhizosphere fungal communities across different plant species (Figure 2C). The ANOSIM confirmed that there was no significant difference in the rhizosphere fungal communities among different plant species (*P* > 0.05; Figure 2C). Mantel tests showed that the rhizosphere fungal communities were significantly positively correlated with the rhizosphere soil variables of pH, TN, and total carbon (TC) (*P* < 0.05; Figure 2D).

### Assembly processes of fungal communities in the rhizosphere

**FIGURE 3:**
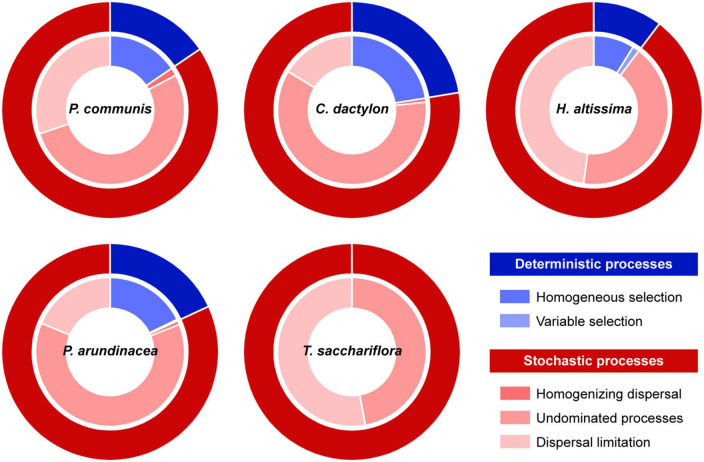
Assembly processes of rhizosphere fungal communities from different plant species. The assembly processes were identified based on two indices of β-nearest taxon index (βNTI) and raup-crick index (RCI). Variable and homogeneous selection belong to deterministic processes; dispersal limitation, homogenizing dispersal, and undominated processes are affiliated to stochastic processes.

In the middle-channel bar, the community assembly of rhizosphere fungi was dominated by the stochastic processes (85.3%), with the contribution of undominated processes and dispersal limitation being 54.5 and 30.1%, respectively (Supplementary Figure 3). Among different plant species, the undominated processes had greater contributions of 52.6, 60.3, 41.9, 62.4, and 47.1% to the fungal community assembly in the rhizosphere of *P. communis*, *C. dactylon*, *H. altissima*, *P. arundinacea*, and *T. sacchariflora* (Figure 3). Compared with undominated processes, dispersal limitation contributed most to the fungal community assembly in the rhizosphere of *H. altissima* (47.8%) and *T. sacchariflora* (52.9%) (Figure 3). Moreover, the homogeneous selection of deterministic processes exhibited indispensable effects on fungal community assembly in the rhizosphere of *P. communis* (15.5%), *C. dactylon* (22.4%), and *P. arundinacea* (17.8%).

**FIGURE 4:**
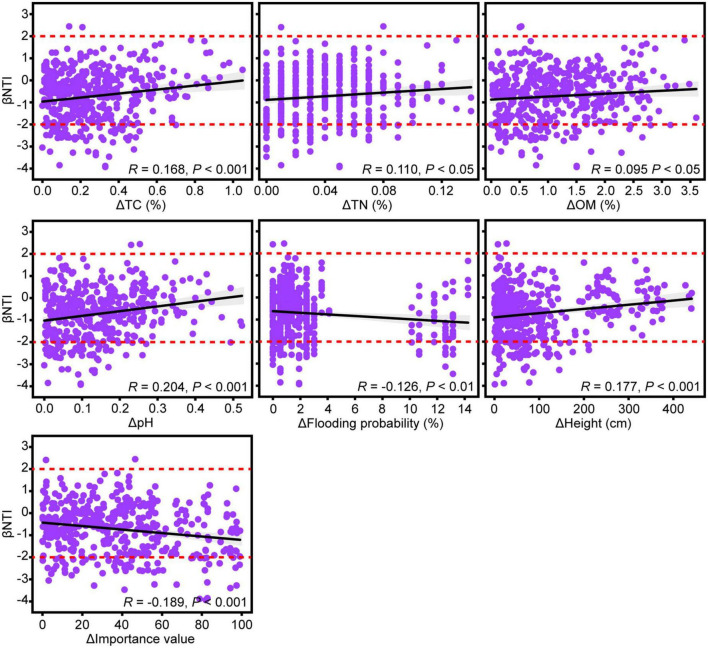
Linear regressions between βNTI of rhizosphere fungal community and changes in soil environmental factors and aboveground plant traits. Only significant relationships are exhibited based on analysis of variance (ANOVA) at 0.05 level of significance. *R* values represent Pearson correlation coefficients. Dotted lines indicate the βNTI significance thresholds of –2 and +2.

The relationships of the βNTI of rhizosphere fungal communities with soil environmental factors and aboveground plant traits were determined to reveal their impacts on the assembly processes of fungal communities. The changes in rhizosphere soil environmental variables of TC, TN, OM, and pH had significant positive correlations with the pairwise comparisons of βNTI (Figure 4). This result suggested that an increasing discrepancy in these soil environmental variables promoted the effect of stochastic processes on the community assembly of rhizosphere fungi. Moreover, the change in aboveground plant traits of height was significantly positively correlated with βNTI (Figure 4). In contrast, the changes in flooding probability of sampling sites and aboveground plant traits of importance value showed significantly negative correlations with βNTI.

### Co-occurrence pattern of rhizosphere fungal community

**FIGURE 5:**
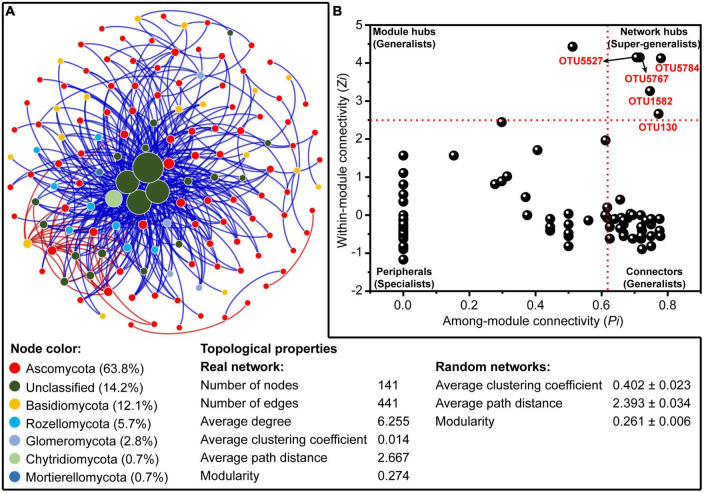
**(A)** The co-occurrence network of rhizosphere fungal community at operational taxonomic unit (OTU) level based on random matrix theory (RMT). The nodes in the network represent OTUs, and the size of each node is proportional to the corresponding node degree (number of connections). The nodes are colored by fungal phylum. The red and blue lines, respectively, indicate positive and negative correlations between any two OTUs. **(B)** The *Zi*-*Pi* plot reveals the distribution of OTUs of rhizosphere fungal community based on the topological properties.

A molecular ecological network composed of 141 nodes with 441 edges (Figure 5A) was constructed based on the RMT to reveal the co-occurrence pattern of the rhizosphere fungal community. The distribution of node degrees for the constructed fungal network showed a scale-free power-law network property (R^2^ = 0.832; Supplementary Figure 4), indicating a non-random structure of the constructed network. Additionally, the values of average path distance (2.667 vs. 2.393) and modularity (0.274 vs. 0.261) were higher than those of the respective randomized network (1,000 Erdös–Réyni), revealing the small-world properties and modular structure of the constructed network. The fungal network was dominated by Ascomycota accounting for 63.8% of the total nodes, followed by unclassified fungi (14.2%) (Figure 5A).

Based on the within-module connectivity (*Zi*) and among-module connectivity (*Pi*) of individual taxa within the constructed network, a total of 5, 1, and 51 nodes were identified as the network hubs, module hubs, and connectors, respectively (Figure 5B and Supplementary Table 1). All of these nodes could be regarded as the keystone species in structuring the rhizosphere fungal communities. Nearly half of the keystone species (47.4%) were affiliated to the unclassified fungi at the class level (Supplementary Table 1). Noteworthily, most connectors showed significant correlations with soil TN, OM, and pH, whereas the network hubs and module hubs were not significantly affected by the measured rhizosphere soil environmental variables and aboveground plant traits (Supplementary Table 2).

## Discussion

In this study, the rhizosphere fungal communities did not show significant differences among different plant species in the mid-channel bar. As the two most abundant fungal genera, *Alternaria* showed a significantly higher relative abundance in the rhizosphere of *C. dactylon* than that of *H. altissima*, while *Epicoccum* showed no significant difference among different plant species (Figure 2A). *Alternaria* genus is a group of ascomycete fungi including both pathogenic and saprophytic species, and can secrete various secondary metabolites with effective mycotoxins (Dang and Lawrence, 2014) and inhibit other microbial growth (Si et al., 2018). This was consistent with our previous study, reporting that the rhizosphere of *C. dactylon* harbored significantly lower relative abundance than that of *H. altissima* in the most abundant bacterial genus *Pseudarthrobacter* (Ye et al., 2022).

Though many studies have reported the species-specific selection of rhizosphere microbial communities by plants through root traits and exudates (Costa et al., 2006; Sweeney et al., 2021; Xiao et al., 2022), soil physicochemical properties were considered to be the primary factors affecting rhizosphere fungal community than plant species type (identity) in some studies (Singh et al., 2007; Chen et al., 2019). Soil can regulate the rhizosphere microbial community; particularly, soil pH and nutrients are regarded as the key factors influencing the structure of the rhizosphere microbial community (Philippot et al., 2013). In this study, the rhizosphere fungal community composition was significantly positively correlated with soil pH and the contents of soil TC and TN (Figure 2D). In contrast, the plant species differentiated in the canopy over, and defense strategies and root exudation might exert more influence on the endosphere than rhizosphere fungal community (Edwards et al., 2015). Moreover, the rhizosphere microorganisms are mostly recruited from the microbial communities in the surrounding soils. Thus, the structure of the rhizosphere microbial community is restricted by the accessible individuals from the species pool of bulk soil (de Boer et al., 2006), making these communities highly dependent on the local environmental conditions around the plant. Notably, the measured plant traits had no significant effect on the rhizosphere fungal community (Figure 2D). This may be attributed to the aboveground plant traits rather than the underground plant traits measured in this study. Previous studies have found that underground plant traits, i.e., root traits, are more important predictors of rhizosphere fungal community composition than aboveground leaf traits (Cantarel et al., 2015; Sweeney et al., 2021). Overall, the compositions of the rhizosphere microbial community are influenced by the intricate associations between the plant species and soil type (Marschner et al., 2001).

Based on the ecological setting in which the assembly processes are engaged, the deterministic and stochastic processes contribute differently to the community assembly (Powell et al., 2015). In this study, the assembly of the rhizosphere fungal community was largely driven by the stochastic processes, wherein the undominated processes contributed the most (Supplementary Figure 3). This result was consistent with the previous study reporting that undominated processes, which means a greater effect of drift in driving community assembly (Stegen et al., 2015), were the most dominant processes for rhizosphere fungal assembly with an average contribution of 79.4–96.3% (Shi et al., 2022). Soil fungi are typically characterized by sporogony, filamentous growth and larger propagules size compared to bacteria (Ingold, 1971; Young, 2007). Spore proliferation helps the fungus release myriad spores against environmental stress (Ingold, 1971). These spores can move around within the environment through physical mediators such as water (Walters et al., 2022). Particularly, the mid-channel bar, a highly permeable sandbar, is more conducive to the widespread distribution of spores through water transmission. The dormancy strategy of fungal communities allows these spores to survive in a new environment and thrive when conditions are favorable (Lennon and Jones, 2011), resulting in negligible environmental effects (i.e., variable selection and homogeneous selection) on the rhizosphere fungal assembly in the mid-channel ecosystem. Therefore, the change in the composition of the rhizosphere fungal community is mainly governed by ecological drift resulting from the fluctuation in population sizes by environmental changes (Stegen et al., 2013).

The differences in the predominance of undominated processes in the assembly processes with plant species could be attributed to the changes in soil properties. Previous studies have reported that soil pH, soil organic carbon, TN, ammonium etc., could significantly affect the assembly processes of soil microbial community (Jiao and Lu, 2020a; Zheng et al., 2021; Zhao et al., 2022). In this study, the βNTI of rhizosphere fungi shared significant associations with the soil pH, OM, TN, and TC. Our previous study has confirmed that the water flooding frequency can change the soil OM accumulation in the mid-channel bar, while low flooding frequency results in the accumulation of nutrients and promotes plant growth (Ye et al., 2022). This phenomenon could be supported by the negative correlation between the βNTI of rhizosphere fungi and water flooding frequency. In contrast, plant growth, affects the enrichment of rhizosphere soil OM by regulating litter inputs and root exudates (Guo et al., 2019). Considering the role of fungi in OM decomposition (Shi et al., 2020), this might explain the influence of soil properties on the assembly of rhizosphere fungal communities. It is noteworthy that the available substrates provided by the plants for rhizosphere fungal communities vary with the growth state of different plant species. This could be demonstrated by the substantial association between the βNTI of rhizosphere fungi and the height of plant species.

Furthermore, the associations between different fungal taxa and the key taxa structuring the assembly of rhizosphere fungal community were explored by the co-occurrence network analysis. In this study, a total of five fungal taxa were identified as the network hubs, suggesting that these taxa had key roles in the rhizosphere fungal community (Deng et al., 2012; Wang et al., 2020). It is noteworthy that these network hubs were almost all unclassified fungi, and had low relative abundances across all rhizosphere soils. Species with low abundance have been defined as rare taxa in numerous studies (Pedrós-Alió, 2012; Shade et al., 2014; Lynch and Neufeld, 2015; Jousset et al., 2017; Jia et al., 2018), which are generally considered to play disproportionate roles in maintaining the structure and function of ecosystems in different habitats (Jia et al., 2018; Wang et al., 2020). Interestingly, these network hubs were largely unaffected by measured abiotic (rhizosphere environmental variables) and biotic factors (aboveground plant traits) (Supplementary Table 2). Overall, these results indicate that these network hubs could remain stable regardless of environmental changes over space and time, which are more likely to be influenced by stochastic dispersal or drift (Stegen et al., 2012; Mo et al., 2018; Zhao et al., 2022).

## Conclusion

This study revealed that the differentiation in plants, in terms of both species and associated aboveground traits, had little effect on the rhizosphere fungal community in the mid-channel bar. In contrast, soil properties (i.e., soil TN, TC, OM, and pH) related to the frequency of water flooding were largely associated with the rhizosphere fungal community. However, the assembly of soil fungal communities in the rhizosphere of all tested plant species was mostly governed by the stochastic processes presented by the undominated processes. Notably, the soil properties had significant impacts on the predominance of undominated processes among different plant species. This regulation was mainly due to the effect of soil properties on the ecological drift intensity of fungi species. Additionally, the effect of the physiological properties of fungal species on the community assembly processes was emphasized. Furthermore, some rare unclassified fungal taxa were found to play an indispensable role in the assembly of rhizosphere fungal communities. These species were almost unaffected by environmental variables, indirectly confirming the effects of stochastic processes. Overall, these provide insights into the assembly and drivers of the rhizosphere fungal community, which is valuable for maintaining the functional stability of a developing ecosystem.

## Data availability statement

The datasets presented in this study can be found in online repositories. The names of the repository/repositories and accession number(s) can be found below: https://www.ncbi.nlm.nih.gov/, PRJNA903773.

## Author contributions

FY, YW, and XY designed the study. FY and XY performed the field work and laboratory work. FY and ZS analyzed the data. FY wrote the manuscript. YW, YH, and JW contributed to the writing and editing. All authors have reviewed the manuscript and approved the submitted version.

## Footnotes

1
http://www.Majorbio.com

2
http://drive5.com/uparse/

3
http://unite.ut.ee/index.php

4
http://ieg4.rccc.ou.edu/mena

5
https://submit.ncbi.nlm.nih.gov/subs/sra/

## References

[B1] Adams R. I. Miletto M. Taylor J. W. Bruns T. D. (2013). Dispersal in microbes: Fungi in indoor air are dominated by outdoor air and show dispersal limitation at short distances. *ISME J.* 7 1262–1273. 10.1038/ismej.2013.28 23426013 PMC3695294

[B2] Bastian M. Heymann S. Jacomy M. (2009). “Gephi: An open source software for exploring and manipulating networks,” in *Proceedings of the international AAAI conference on weblogs and social media*, (San Jose, CA: AAAI).

[B3] Bolger A. M. Lohse M. Usadel B. (2014). Trimmomatic: A flexible trimmer for Illumina sequence data. *Bioinformatics* 30 2114–2120. 10.1093/bioinformatics/btu170 24695404 PMC4103590

[B4] Brundrett M. C. Tedersoo L. (2018). Evolutionary history of mycorrhizal symbioses and global host plant diversity. *New Phytol.* 220 1108–1115. 10.1111/nph.14976 29355963

[B5] Cantarel A. A. M. Pommier T. Desclos-Theveniau M. Diquélou S. Dumont M. Grassein F. (2015). Using plant traits to explain plant–microbe relationships involved in nitrogen acquisition. *Ecology* 96 788–799. 10.1890/13-2107.1 26236874

[B6] Chen L. Xin X. Zhang J. Redmile-Gordon M. Nie G. Wang Q. (2019). Soil characteristics overwhelm cultivar effects on the structure and assembly of root-associated microbiomes of modern maize. *Pedosphere* 29 360–373. 10.1016/S1002-0160(17)60370-9

[B7] Costa R. Götz M. Mrotzek N. Lottmann J. Berg G. Smalla K. (2006). Effects of site and plant species on rhizosphere community structure as revealed by molecular analysis of microbial guilds. *FEMS Microbiol. Ecol.* 56 236–249. 10.1111/j.1574-6941.2005.00026.x 16629753

[B8] Dang H. X. Lawrence C. B. (2014). “Alternaria comparative genomics: The secret life of rots,” in *Genomics of plant-associated fungi and oomycetes: Dicot pathogens*, eds Dean R. A. Lichens-Park A. Kole C. (Berlin: Springer Berlin Heidelberg), 45–63.

[B9] de Boer W. Kowalchuk G. A. van Veen J. A. (2006). ‘Root-food’ and the rhizosphere microbial community composition. *New Phytol.* 170 3–6. 10.1111/j.1469-8137.2006.01674.x 16539597

[B10] Deng Y. Jiang Y. H. Yang Y. He Z. Luo F. Zhou J. (2012). Molecular ecological network analyses. *BMC Bioinform.* 13:113. 10.1186/1471-2105-13-113 22646978 PMC3428680

[B11] Dini-Andreote F. Stegen J. C. van Elsas J. D. Salles J. F. (2015). Disentangling mechanisms that mediate the balance between stochastic and deterministic processes in microbial succession. *Proc. Natl. Acad. Sci. U.S.A.* 112 E1326–E1332. 10.1073/pnas.1414261112 25733885 PMC4371938

[B12] Edwards J. Johnson C. Santos-Medellín C. Lurie E. Podishetty N. K. Bhatnagar S. (2015). Structure, variation, and assembly of the root-associated microbiomes of rice. *Proc. Natl. Acad. Sci. U.S.A.* 112 E911–E920. 10.1073/pnas.1414592112 25605935 PMC4345613

[B13] Guo N. Degen A. A. Deng B. Shi F. Bai Y. Zhang T. (2019). Changes in vegetation parameters and soil nutrients along degradation and recovery successions on alpine grasslands of the Tibetan plateau. *Agr. Ecosyst. Environ.* 284:106593. 10.1016/j.agee.2019.106593

[B14] Hong Y. Zhou Q. Hao Y. Huang A. C. (2022). Crafting the plant root metabolome for improved microbe-assisted stress resilience. *New Phytol.* 234 1945–1950. 10.1111/nph.17908 34877653

[B15] Hooke J. M. Yorke L. (2011). Channel bar dynamics on multi-decadal timescales in an active meandering river. *Earth Surf. Proc. Land.* 36 1910–1928. 10.1002/esp.2214

[B16] Ingold C. T. (1971). *Fungal spores: Their liberation and dispersal.* Oxford: Clarendon Press.

[B17] Ji M. Kong W. Stegen J. Yue L. Wang F. Dong X. (2020). Distinct assembly mechanisms underlie similar biogeographical patterns of rare and abundant bacteria in Tibetan Plateau grassland soils. *Environ. Microbiol.* 22 2261–2272. 10.1111/1462-2920.14993 32216022

[B18] Jia X. Dini-Andreote F. Falcão Salles J. (2018). Community assembly processes of the microbial rare biosphere. *Trends Microbiol.* 26 738–747. 10.1016/j.tim.2018.02.011 29550356

[B19] Jiao S. Lu Y. (2020b). Soil pH and temperature regulate assembly processes of abundant and rare bacterial communities in agricultural ecosystems. *Environ. Microbiol.* 22 1052–1065. 10.1111/1462-2920.14815 31599105

[B20] Jiao S. Lu Y. (2020a). Abundant fungi adapt to broader environmental gradients than rare fungi in agricultural fields. *Glob. Change Biol.* 26 4506–4520. 10.1111/gcb.15130 32324306

[B21] Jousset A. Bienhold C. Chatzinotas A. Gallien L. Gobet A. Kurm V. (2017). Where less may be more: How the rare biosphere pulls ecosystems strings. *ISME J.* 11 853–862. 10.1038/ismej.2016.174 28072420 PMC5364357

[B22] Lennon J. T. Jones S. E. (2011). Microbial seed banks: The ecological and evolutionary implications of dormancy. *Nat. Rev. Microbiol.* 9 119–130. 10.1038/nrmicro2504 21233850

[B23] Lou Y. Mei X. Dai Z. Wang J. Wei W. (2018). Evolution of the mid-channel bars in the middle and lower reaches of the Changjiang (Yangtze) River from 1989 to 2014 based on the Landsat satellite images: Impact of the Three Gorges Dam. *Environ. Earth Sci.* 77 394. 10.1007/s12665-018-7576-2

[B24] Lynch M. D. J. Neufeld J. D. (2015). Ecology and exploration of the rare biosphere. *Nat. Rev. Microbiol.* 13 217–229. 10.1038/nrmicro3400 25730701

[B25] Ma B. Wang H. Dsouza M. Lou J. He Y. Dai Z. (2016). Geographic patterns of co-occurrence network topological features for soil microbiota at continental scale in eastern China. *ISME J.* 10 1891–1901. 10.1038/ismej.2015.261 26771927 PMC5029158

[B26] Marschner P. Yang C. H. Lieberei R. Crowley D. E. (2001). Soil and plant specific effects on bacterial community composition in the rhizosphere. *Soil Biol. Biochem.* 33 1437–1445. 10.1016/S0038-0717(01)00052-9

[B27] Mo Y. Zhang W. Yang J. Lin Y. Yu Z. Lin S. (2018). Biogeographic patterns of abundant and rare bacterioplankton in three subtropical bays resulting from selective and neutral processes. *ISME J.* 12 2198–2210. 10.1038/s41396-018-0153-6 29880912 PMC6092436

[B28] Pedrós-Alió C. (2012). The rare bacterial biosphere. *Annu. Rev. Mar. Sci.* 4 449–466. 10.1146/annurev-marine-120710-100948 22457983

[B29] Philippot L. Raaijmakers J. M. Lemanceau P. van der Putten W. H. (2013). Going back to the roots: The microbial ecology of the rhizosphere. *Nat. Rev. Microbiol.* 11 789–799. 10.1038/nrmicro3109 24056930

[B30] Potts L. D. Douglas A. Perez Calderon L. J. Anderson J. A. Witte U. Prosser J. I. (2022). Chronic environmental perturbation influences microbial community assembly patterns. *Environ. Sci. Technol.* 56 2300–2311. 10.1021/acs.est.1c05106 35103467 PMC9007448

[B31] Powell J. R. Karunaratne S. Campbell C. D. Yao H. Robinson L. Singh B. K. (2015). Deterministic processes vary during community assembly for ecologically dissimilar taxa. *Nat. Commun.* 6:8444. 10.1038/ncomms9444 26436640 PMC4600744

[B32] Qin Q. Wang Y. Qiu C. Zheng D. Liu Y. (2022). Wildfire drives the transition from deterministic- to stochastic-dominated community assembly of abundant bacterial in forest soils. *CATENA* 215:106290. 10.1016/j.catena.2022.106290

[B33] Shade A. Jones S. E. Caporaso J. G. Handelsman J. Knight R. Fierer N. (2014). Conditionally rare taxa disproportionately contribute to temporal changes in microbial diversity. *mBio* 5 e1371–e1314. 10.1128/mBio.01371-14 25028427 PMC4161262

[B34] Shi Y. Dang K. Dong Y. Feng M. Wang B. Li J. (2020). Soil fungal community assembly processes under long-term fertilization. *Eur. J. Soil Sci.* 71 716–726. 10.1111/ejss.12902

[B35] Shi Y. Zhang K. Ma T. Zhang Z. Li P. Xing Z. (2022). Foliar herbivory reduces rhizosphere fungal diversity and destabilizes the co-occurrence network. *Front. Microbiol.* 13:846332. 10.3389/fmicb.2022.846332 35350618 PMC8957981

[B36] Si P. Shao W. Yu H. Yang X. Gao D. Qiao X. (2018). Rhizosphere microenvironments of eight common deciduous fruit trees were shaped by microbes in Northern China. *Front. Microbiol.* 9:3147. 10.3389/fmicb.2018.03147 30619213 PMC6305578

[B37] Singh B. K. Munro S. Potts J. M. Millard P. (2007). Influence of grass species and soil type on rhizosphere microbial community structure in grassland soils. *Appl. Soil Ecol.* 36 147–155. 10.1016/j.apsoil.2007.01.004

[B38] Stegen J. C. Lin X. Fredrickson J. K. Chen X. Kennedy D. W. Murray C. J. (2013). Quantifying community assembly processes and identifying features that impose them. *ISME J.* 7 2069–2079. 10.1038/ismej.2013.93 23739053 PMC3806266

[B39] Stegen J. C. Lin X. Fredrickson J. K. Konopka A. E. (2015). Estimating and mapping ecological processes influencing microbial community assembly. *Front. Microbiol.* 6:370. 10.3389/fmicb.2015.00370 25983725 PMC4416444

[B40] Stegen J. C. Lin X. Konopka A. E. Fredrickson J. K. (2012). Stochastic and deterministic assembly processes in subsurface microbial communities. *ISME J.* 6 1653–1664. 10.1038/ismej.2012.22 22456445 PMC3498916

[B41] Sweeney C. J. de Vries F. T. van Dongen B. E. Bardgett R. D. (2021). Root traits explain rhizosphere fungal community composition among temperate grassland plant species. *New Phytol.* 229 1492–1507. 10.1111/nph.16976 33006139

[B42] Tabacchi E. Steiger J. Corenblit D. Monaghan M. T. Planty-Tabacchi A.-M. (2009). Implications of biological and physical diversity for resilience and resistance patterns within Highly Dynamic River Systems. *Aquat. Sci.* 71:279. 10.1007/s00027-009-9195-1

[B43] Ter Braak C. J. F. Šmilauer P. (2012). *Canoco reference manual and user’s guide: Software of ordination, version 5.0.* Ithaca, NY: Microcomputer Power.

[B44] Tonkin J. D. Merritt D. M. Olden J. D. Reynolds L. V. Lytle D. A. (2018). Flow regime alteration degrades ecological networks in riparian ecosystems. *Nat. Ecol. Evol.* 2 86–93. 10.1038/s41559-017-0379-0 29180707

[B45] Walters K. E. Capocchi J. K. Albright M. B. N. Hao Z. Brodie E. L. Martiny J. B. H. (2022). Routes and rates of bacterial dispersal impact surface soil microbiome composition and functioning. *ISME J.* 16 2295–2304. 10.1038/s41396-022-01269-w 35778440 PMC9477824

[B46] Wan W. Grossart H. P. He D. Yuan W. Yang Y. (2021). Stronger environmental adaptation of rare rather than abundant bacterioplankton in response to dredging in eutrophic Lake Nanhu (Wuhan, China). *Water Res.* 190:116751. 10.1016/j.watres.2020.116751 33348071

[B47] Wang F. Li Y. T. Liu Y. Li M. (2015). Evolution of multi-bar in Shashi reach before and after Three Gorges Reservoir impoundment. *J. Sediment Res.* 4 1–6. (In Chinese).

[B48] Wang Y. Ye F. Wu S. Wu J. Yan J. Xu K. (2020). Biogeographic pattern of bacterioplanktonic community and potential function in the Yangtze River: Roles of abundant and rare taxa. *Sci. Total Environ.* 747:141335. 10.1016/j.scitotenv.2020.141335 32795800

[B49] Wei G. Li M. Shi W. Tian R. Chang C. Wang Z. (2020). Similar drivers but different effects lead to distinct ecological patterns of soil bacterial and archaeal communities. *Soil Biol. Biochem.* 144:107759. 10.1016/j.soilbio.2020.107759

[B50] Wen Z. Yang H. Zhang C. Shao G. Wu S. (2020). Remotely sensed mid-channel bar dynamics in downstream of the Three Gorges Dam, China. *Remote Sensing* 12:409. 10.3390/rs12030409

[B51] Xiao X. Wang J. L. Li J. J. Li X. L. Dai X. J. Shen R. F. (2022). Distinct patterns of rhizosphere microbiota associated with rice genotypes differing in aluminum tolerance in an acid sulfate soil. *Front. Microbiol.* 13:933722. 10.3389/fmicb.2022.933722 35783428 PMC9247542

[B52] Ye F. Hong Y. Wu J. Yi X. Op den Camp H. J. M. Moore S. S. (2022). Succession of soil microbial community in a developing mid-channel bar: The role of environmental disturbance and plant community. *Front. Microbiol.* 13:970529. 10.3389/fmicb.2022.970529 36060763 PMC9428583

[B53] Ye F. Wang X. Wang Y. Wu S. Wu J. Hong Y. (2021). Different pioneer plant species have similar rhizosphere microbial communities. *Plant Soil* 464 165–181. 10.1007/s11104-021-04952-7

[B54] Young K. D. (2007). Bacterial morphology: Why have different shapes? *Curr. Opin. Microbiol.* 10 596–600. 10.1016/j.mib.2007.09.009 17981076 PMC2169503

[B55] Zhalnina K. Louie K. B. Hao Z. Mansoori N. da Rocha U. N. Shi S. (2018). Dynamic root exudate chemistry and microbial substrate preferences drive patterns in rhizosphere microbial community assembly. *Nat. Microbiol.* 3 470–480. 10.1038/s41564-018-0129-3 29556109

[B56] Zhang L. Delgado-Baquerizo M. Shi Y. Liu X. Yang Y. Chu H. (2021). Co-existing water and sediment bacteria are driven by contrasting environmental factors across glacier-fed aquatic systems. *Water Res.* 198:117139. 10.1016/j.watres.2021.117139 33895591

[B57] Zhao Z. Ma Y. Feng T. Kong X. Wang Z. Zheng W. (2022). Assembly processes of abundant and rare microbial communities in orchard soil under a cover crop at different periods. *Geoderma* 406:115543. 10.1016/j.geoderma.2021.115543

[B58] Zheng W. Zhao Z. Lv F. Wang R. Wang Z. Zhao Z. (2021). Assembly of abundant and rare bacterial and fungal sub-communities in different soil aggregate sizes in an apple orchard treated with cover crop and fertilizer. *Soil Biol. Biochem.* 156:108222. 10.1016/j.soilbio.2021.108222

[B59] Zhou J. Deng Y. Luo F. He Z. Yang Y. (2011). Phylogenetic molecular ecological network of soil microbial communities in response to elevated CO_2_. *mBio* 2 e122–e111. 10.1128/mBio.00122-11 21791581 PMC3143843

