# Supplementary material for: Stochastic processes drive the soil fungal communities in a developing mid-channel bar
Source: Front Microbiol. 2023 Feb 6;14:1104297. doi: 10.3389/fmicb.2023.1104297 (PMC9939660; doi:10.3389/fmicb.2023.1104297)
Supplement: Supplementary file 1 [file Data_Sheet_1.docx]

**Supplementary materials**

**Stochastic processes drive the soil fungal communities in a developing mid-channel bar**

**Fei Ye^1^, Yiguo Hong^1^, Xuemei Yi^2^, Zhaohong Sun^1^, Jiapeng Wu^1^, Yu Wang^1,*^**

^1^ Institute of Environmental Research at Greater Bay Area, Key Laboratory for Water Quality and Conservation of the Pearl River Delta, Ministry of Education, Guangzhou University, Guangzhou 510006, China

^2^ Chongqing Institute of Green and Intelligent Technology, Chinese Academy of Sciences, Chongqing 401122, China

***Corresponding author:**

Yu Wang: wangyu@gzhu.edu.cn

**Supplementary Table S1** Lists of keystone species in the co-occurrence network.

| Keystone | Role | Phylum | Class | Order | Family | Genus |
| --- | --- | --- | --- | --- | --- | --- |
| OTU5784 | Network hubs | Unclassified | Unclassified | Unclassified | Unclassified | Unclassified |
| OTU5527 |  | Unclassified | Unclassified | Unclassified | Unclassified | Unclassified |
| OTU5767 |  | Unclassified | Unclassified | Unclassified | Unclassified | Unclassified |
| OTU1582 |  | Chytridiomycota | Unclassified | Unclassified | Unclassified | Unclassified |
| OTU130 |  | Unclassified | Unclassified | Unclassified | Unclassified | Unclassified |
| OTU5911 | Module hubs | Ascomycota | Sordariomycetes | Xylariales | Microdochiaceae | Microdochium |
| OTU5408 | Connecters | Ascomycota | Dothideomycetes | Pleosporales | Unclassified | Unclassified |
| OTU3562 |  | Unclassified | Unclassified | Unclassified | Unclassified | Unclassified |
| OTU3850 |  | Ascomycota | Sordariomycetes | Glomerellales | Plectosphaerellaceae | Acrostalagmus |
| OTU2926 |  | Unclassified | Unclassified | Unclassified | Unclassified | Unclassified |
| OTU1460 |  | Unclassified | Unclassified | Unclassified | Unclassified | Unclassified |
| OTU4807 |  | Ascomycota | Sordariomycetes | Hypocreales | Clavicipitaceae | Metarhizium |
| OTU4638 |  | Ascomycota | Dothideomycetes | Pleosporales | Thyridariaceae | Unclassified |
| OTU2421 |  | Ascomycota | Sordariomycetes | Unclassified | Unclassified | Unclassified |
| OTU1482 |  | Glomeromycota | Glomeromycetes | Diversisporales | Diversisporaceae | Unclassified |
| OTU2584 |  | Glomeromycota | Paraglomeromycetes | GS24 | Unclassified | Unclassified |
| OTU4654 |  | Ascomycota | Dothideomycetes | Pleosporales | Thyridariaceae | Roussoella |
| OTU3184 |  | Unclassified | Unclassified | Unclassified | Unclassified | Unclassified |
| OTU4484 |  | Unclassified | Unclassified | Unclassified | Unclassified | Unclassified |
| OTU400 |  | Ascomycota | Eurotiomycetes | Eurotiales | Aspergillaceae | Penicillium |
| OTU4295 |  | Ascomycota | Sordariomycetes | Hypocreales | Clavicipitaceae | Collarina |
| OTU5003 |  | Ascomycota | Sordariomycetes | Hypocreales | Clavicipitaceae | Metarhizium |
| OTU4389 |  | Rozellomycota | Unclassified | Unclassified | Unclassified | Unclassified |
| OTU4710 |  | Rozellomycota | Unclassified | Unclassified | Unclassified | Unclassified |
| OTU2209 |  | Rozellomycota | Unclassified | Unclassified | Unclassified | Unclassified |
| OTU1117 |  | Ascomycota | Unclassified | Unclassified | Unclassified | Unclassified |
| OTU5076 |  | Ascomycota | Sordariomycetes | Hypocreales | Nectriaceae | Fusicolla |
| OTU2513 |  | Ascomycota | Dothideomycetes | Capnodiales | Mycosphaerellaceae | Zymoseptoria |
| OTU4396 |  | Unclassified | Unclassified | Unclassified | Unclassified | Unclassified |
| OTU1895 |  | Rozellomycota | Unclassified | Unclassified | Unclassified | Unclassified |
| OTU774 |  | Unclassified | Unclassified | Unclassified | Unclassified | Unclassified |
| OTU719 |  | Rozellomycota | Unclassified | Unclassified | Unclassified | Unclassified |
| OTU1693 |  | Unclassified | Unclassified | Unclassified | Unclassified | Unclassified |
| OTU3455 |  | Ascomycota | Leotiomycetes | Helotiales | Sclerotiniaceae | Ciboria |
| OTU4465 |  | Ascomycota | Leotiomycetes | Helotiales | Dermateaceae | Patinella |
| OTU1764 |  | Mortierellomycota | Unclassified | Unclassified | Unclassified | Unclassified |
| OTU66 |  | Ascomycota | Sordariomycetes | Microascales | Microascaceae | Scedosporium |
| OTU1714 |  | Unclassified | Unclassified | Unclassified | Unclassified | Unclassified |
| OTU5773 |  | Ascomycota | Dothideomycetes | Pleosporales | Unclassified | Unclassified |
| OTU1120 |  | Ascomycota | Dothideomycetes | Pleosporales | Unclassified | Unclassified |
| OTU331 |  | Unclassified | Unclassified | Unclassified | Unclassified | Unclassified |
| OTU2141 |  | Rozellomycota | Unclassified | Unclassified | Unclassified | Unclassified |
| OTU3985 |  | Rozellomycota | Unclassified | Unclassified | Unclassified | Unclassified |
| OTU1330 |  | Ascomycota | Sordariomycetes | Hypocreales | Hypocreaceae | Trichoderma |
| OTU781 |  | Unclassified | Unclassified | Unclassified | Unclassified | Unclassified |
| OTU136 |  | Ascomycota | Sordariomycetes | Hypocreales | Stachybotryaceae | Alfaria |
| OTU3580 |  | Ascomycota | Unclassified | Unclassified | Unclassified | Unclassified |
| OTU2892 |  | Glomeromycota | Glomeromycetes | Diversisporales | Diversisporaceae | Diversispora |
| OTU2483 |  | Ascomycota | Eurotiomycetes | Eurotiales | Aspergillaceae | Penicillium |
| OTU3163 |  | Ascomycota | Sordariomycetes | Hypocreales | Nectriaceae | Unclassified |
| OTU530 |  | Basidiomycota | Microbotryomycetes | Microbotryales | Ustilentylomataceae | Unclassified |
| OTU2037 |  | Ascomycota | Dothideomycetes | Pleosporales | Unclassified | Unclassified |
| OTU4118 |  | Ascomycota | Sordariomycetes | Coniochaetales | Coniochaetaceae | Lecythophora |
| OTU3356 |  | Ascomycota | Sordariomycetes | Chaetosphaeriales | Chaetosphaeriaceae | Gonytrichum |
| OTU88 |  | Ascomycota | Sordariomycetes | Xylariales | Xylariaceae | Creosphaeria |
| OTU1731 |  | Rozellomycota | Unclassified | Unclassified | Unclassified | Unclassified |
| OTU2331 |  | Ascomycota | Dothideomycetes | Pleosporales | Unclassified | Unclassified |

**Supplementary Table S2** Relationships of keystone species with soil environmental factors and aboveground plant traits.

| Keystone | Role | TC | TN | TS | OM | pH | Flooding probability | Coverage | Height | Richness | Importance value |
| --- | --- | --- | --- | --- | --- | --- | --- | --- | --- | --- | --- |
|  |  | Pearson correlation coefficient *R* | | | | | | | | | |
| OTU5784 | Network hubs | ‒0.084 | ‒0.140 | ‒0.051 | ‒0.212 | 0.213 | 0.160 | ‒0.236 | ‒0.257 | **‒0.417** | 0.172 |
| OTU5527 |  | -0.127 | -0.235 | 0.123 | -0.336 | 0.233 | 0.105 | -0.308 | -0.18 | **-0.457** | 0.304 |
| OTU5767 |  | -0.274 | -0.265 | -0.001 | **-0.363** | 0.326 | 0.104 | -0.331 | -0.221 | **-0.560** | **0.365** |
| OTU1582 |  | -0.129 | -0.28 | 0.043 | -0.3 | 0.2 | 0.15 | -0.219 | -0.091 | -0.199 | -0.001 |
| OTU130 |  | -0.205 | -0.259 | -0.146 | **-0.381** | 0.34 | -0.252 | -0.222 | -0.183 | -0.22 | -0.125 |
| OTU5911 | Module hubs | -0.051 | -0.234 | 0.122 | -0.306 | 0.226 | 0.146 | -0.252 | -0.099 | **-0.389** | -0.141 |
| OTU5408 | Connecters | 0.078 | 0.298 | 0.012 | 0.28 | -0.343 | 0.145 | 0.299 | -0.017 | 0.192 | 0.018 |
| OTU3562 |  | 0.057 | 0.262 | 0.355 | 0.32 | -0.241 | 0.133 | 0.322 | -0.041 | 0.324 | -0.136 |
| OTU3850 |  | **0.406** | **0.457** | 0.25 | **0.536** | **-0.410** | 0.176 | 0.266 | -0.104 | **0.423** | -0.235 |
| OTU2926 |  | 0.131 | **0.459** | 0.296 | **0.439** | **-0.431** | 0.142 | 0.352 | 0.032 | 0.287 | -0.173 |
| OTU1460 |  | **0.419** | **0.589** | 0.251 | **0.548** | **-0.583** | 0.154 | 0.129 | -0.054 | 0.317 | -0.36 |
| OTU4807 |  | **0.452** | **0.391** | 0.214 | **0.449** | **-0.401** | 0.271 | 0.251 | 0.04 | **0.405** | -0.06 |
| OTU4638 |  | **0.372** | **0.639** | 0.026 | **0.659** | **-0.683** | 0.151 | 0.265 | 0.144 | 0.278 | -0.264 |
| OTU2421 |  | 0.17 | 0.169 | **0.425** | 0.127 | -0.326 | 0.118 | -0.062 | -0.084 | 0.11 | -0.281 |
| OTU1482 |  | 0.32 | **0.496** | 0.214 | **0.510** | **-0.418** | 0.161 | **0.505** | 0.239 | 0.249 | -0.032 |
| OTU2584 |  | 0.116 | **0.495** | 0.216 | **0.491** | -0.358 | 0.087 | 0.296 | -0.147 | 0.239 | -0.31 |
| OTU4654 |  | 0.036 | 0.347 | 0.256 | 0.358 | -0.342 | 0.109 | 0.347 | -0.095 | 0.328 | -0.169 |
| OTU3184 |  | **0.413** | **0.623** | 0.131 | **0.558** | **-0.404** | 0.086 | 0.34 | 0.356 | 0.185 | -0.09 |
| OTU4484 |  | 0.09 | **0.477** | 0.182 | **0.373** | **-0.493** | 0.088 | **0.480** | 0.336 | 0.239 | -0.003 |
| OTU400 |  | **0.465** | **0.655** | -0.062 | 0.**549** | **-0.566** | 0.144 | 0.329 | **0.367** | 0.198 | -0.052 |
| OTU4295 |  | 0.197 | 0.33 | **0.374** | **0.388** | -0.232 | 0.11 | 0.286 | 0 | 0.352 | -0.17 |
| OTU5003 |  | **0.512** | 0.201 | -0.045 | 0.181 | -0.286 | 0.16 | -0.1 | -0.08 | 0.242 | -0.081 |
| OTU4389 |  | 0.008 | 0.332 | 0.322 | 0.346 | **-0.462** | 0.135 | 0.225 | -0.031 | 0.3 | -0.235 |
| OTU4710 |  | 0.359 | **0.419** | 0.285 | **0.460** | **-0.374** | 0.166 | 0.225 | -0.046 | **0.364** | -0.199 |
| OTU2209 |  | 0.174 | **0.467** | 0.3 | **0.463** | **-0.411** | 0.131 | 0.31 | 0.031 | 0.246 | -0.161 |
| OTU1117 |  | 0.091 | 0.314 | -0.022 | 0.27 | -0.246 | 0.076 | 0.351 | -0.035 | 0.09 | -0.037 |
| OTU5076 |  | **0.456** | 0.303 | 0.004 | **0.431** | -0.029 | 0.042 | -0.083 | 0.036 | 0.035 | -0.189 |
| OTU2513 |  | 0.134 | 0.141 | -0.112 | 0.257 | -0.073 | 0.013 | 0.039 | -0.06 | **0.425** | -0.227 |
| OTU4396 |  | -0.03 | **0.416** | -0.07 | **0.407** | -0.302 | -0.017 | **0.456** | -0.033 | 0.219 | -0.196 |
| OTU1895 |  | 0.045 | **0.452** | -0.006 | **0.394** | **-0.523** | 0.088 | 0.33 | 0.201 | 0.136 | -0.068 |
| OTU774 |  | 0.285 | **0.509** | 0.253 | **0.495** | -0.354 | 0.117 | 0.256 | 0.137 | 0.203 | -0.176 |
| OTU719 |  | 0.101 | **0.446** | 0.052 | **0.434** | **-0.463** | 0.08 | 0.221 | 0.18 | 0.092 | -0.127 |
| OTU1693 |  | -0.015 | 0.29 | 0.29 | 0.308 | **-0.423** | 0.13 | 0.305 | -0.005 | 0.309 | -0.154 |
| OTU3455 |  | 0.173 | 0.264 | **0.372** | 0.345 | -0.189 | 0.144 | 0.299 | 0.015 | 0.334 | -0.111 |
| OTU4465 |  | 0.17 | **0.421** | 0.157 | **0.442** | **-0.378** | 0.158 | 0.277 | -0.057 | 0.277 | -0.174 |
| OTU1764 |  | 0.165 | 0.227 | 0.06 | 0.184 | -0.066 | -0.011 | -0.119 | 0.051 | 0.037 | -0.229 |
| OTU66 |  | -0.006 | 0.303 | 0.089 | 0.058 | -0.182 | **-0.410** | **0.417** | **0.531** | 0.01 | 0.323 |
| OTU1714 |  | 0.051 | 0.266 | 0.187 | 0.305 | **-0.589** | 0.185 | -0.04 | 0.177 | 0.155 | -0.28 |
| OTU5773 |  | 0.309 | **0.481** | **0.376** | **0.525** | **-0.390** | 0.166 | 0.15 | -0.019 | 0.221 | -0.226 |
| OTU1120 |  | -0.007 | -0.003 | 0.199 | -0.158 | 0.207 | -0.038 | -0.001 | 0.249 | 0.168 | 0.009 |
| OTU331 |  | 0.086 | **0.463** | 0.141 | **0.430** | **-0.541** | 0.125 | 0.321 | 0.118 | 0.208 | -0.136 |
| OTU2141 |  | 0.158 | **.426** | 0.09 | **0.457** | **0-.576** | 0.117 | 0.139 | 0.048 | 0.182 | -0.228 |
| OTU3985 |  | **0.369** | **0.436** | 0.263 | **0.545** | **-0.393** | 0.17 | 0.179 | -0.035 | **0.375** | -0.269 |
| OTU1330 |  | **0.471** | **0.580** | 0.247 | **0.650** | **-0.403** | 0.16 | 0.272 | 0.195 | 0.359 | -0.223 |
| OTU781 |  | 0.127 | **0.505** | 0.231 | **0.387** | **-0.521** | 0.11 | **0.442** | 0.339 | 0.243 | -0.029 |
| OTU136 |  | 0.198 | **0.436** | -0.023 | 0.334 | -0.149 | -0.015 | 0.03 | 0.007 | 0.045 | -0.258 |
| OTU3580 |  | **0.383** | **0.363** | 0.138 | **0.377** | **-0.604** | 0.205 | 0.006 | -0.064 | 0.35 | -0.259 |
| OTU2892 |  | 0.199 | **0.426** | -0.02 | 0.352 | -0.34 | 0.068 | -0.026 | -0.074 | 0.132 | -0.303 |
| OTU2483 |  | 0.172 | **0.364** | 0.336 | **0.420** | -0.298 | 0.165 | 0.355 | 0.018 | **0.366** | -0.134 |
| OTU3163 |  | **0.486** | **0.492** | 0.18 | **0.502** | **-0.486** | 0.212 | 0.302 | 0.237 | **0.468** | -0.111 |
| OTU530 |  | 0.131 | **0.407** | 0.124 | **0.438** | -0.36 | 0.142 | **0.373** | -0.051 | 0.206 | -0.076 |
| OTU2037 |  | 0.268 | 0.319 | 0.289 | **0.362** | -0.346 | 0.207 | 0.313 | -0.057 | **0.433** | -0.086 |
| OTU4118 |  | 0.123 | 0.35 | 0.045 | 0.214 | -0.349 | 0.011 | **0.436** | **0.599** | -0.005 | 0.195 |
| OTU3356 |  | -0.001 | 0.145 | 0.356 | 0.204 | -0.122 | 0.098 | 0.332 | -0.042 | 0.313 | -0.072 |
| OTU88 |  | -0.058 | **0.443** | -0.027 | 0.24 | **-0.361** | -0.151 | **0.524** | **0.384** | 0.158 | 0.018 |
| OTU1731 |  | **0.387** | **0.424** | 0.043 | **0.547** | -0.303 | 0.107 | 0.113 | 0.022 | 0.201 | -0.215 |
| OTU2331 |  | 0.041 | 0.293 | -0.125 | 0.323 | -0.16 | 0.029 | 0.341 | -0.059 | 0.237 | -0.121 |

Note: *S*tatistically significant relationships are bolded based on 0.05 level of significance.

**Supplementary figures**


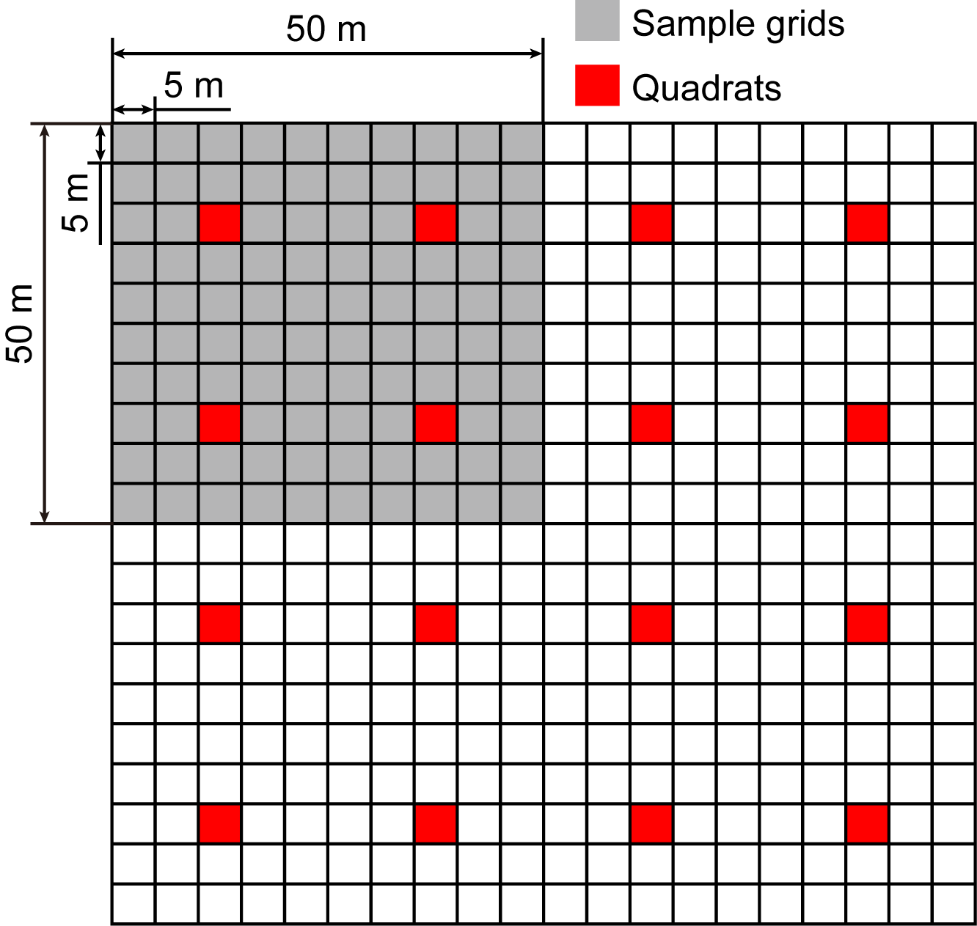


**Supplementary Figure S1.** Schematic diagram of quadrat setup. 5 × 5 m quadrats regularly distribute in 50 × 50 m sample grids with a 25-m interval between each quadrat.


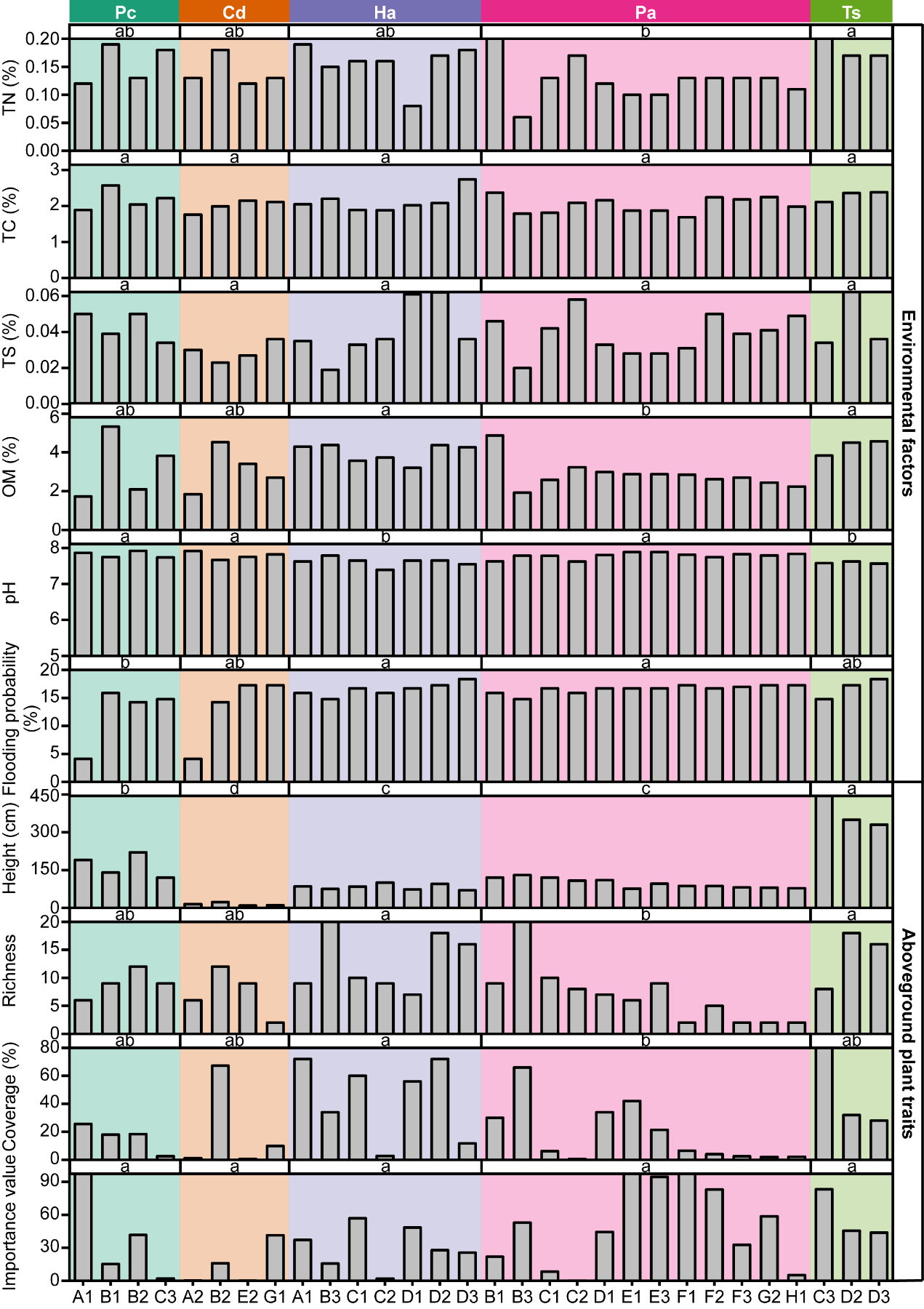


**Supplementary Figure S2.** Rhizosphere environmental conditions and aboveground traits of different plant species in each sampling quadrat. The combination of capital letter and number represents the identity of each sampling quadrat, where capital letters and numbers indicate the positions of the sampling quadrats in the transverse and longitudinal directions, respectively. Different lowercase letters indicate significant differences among different plant species at 0.05 level of significance based on one-way analysis of variance (ANOVA) with least significant difference (LSD) multiple comparisons.


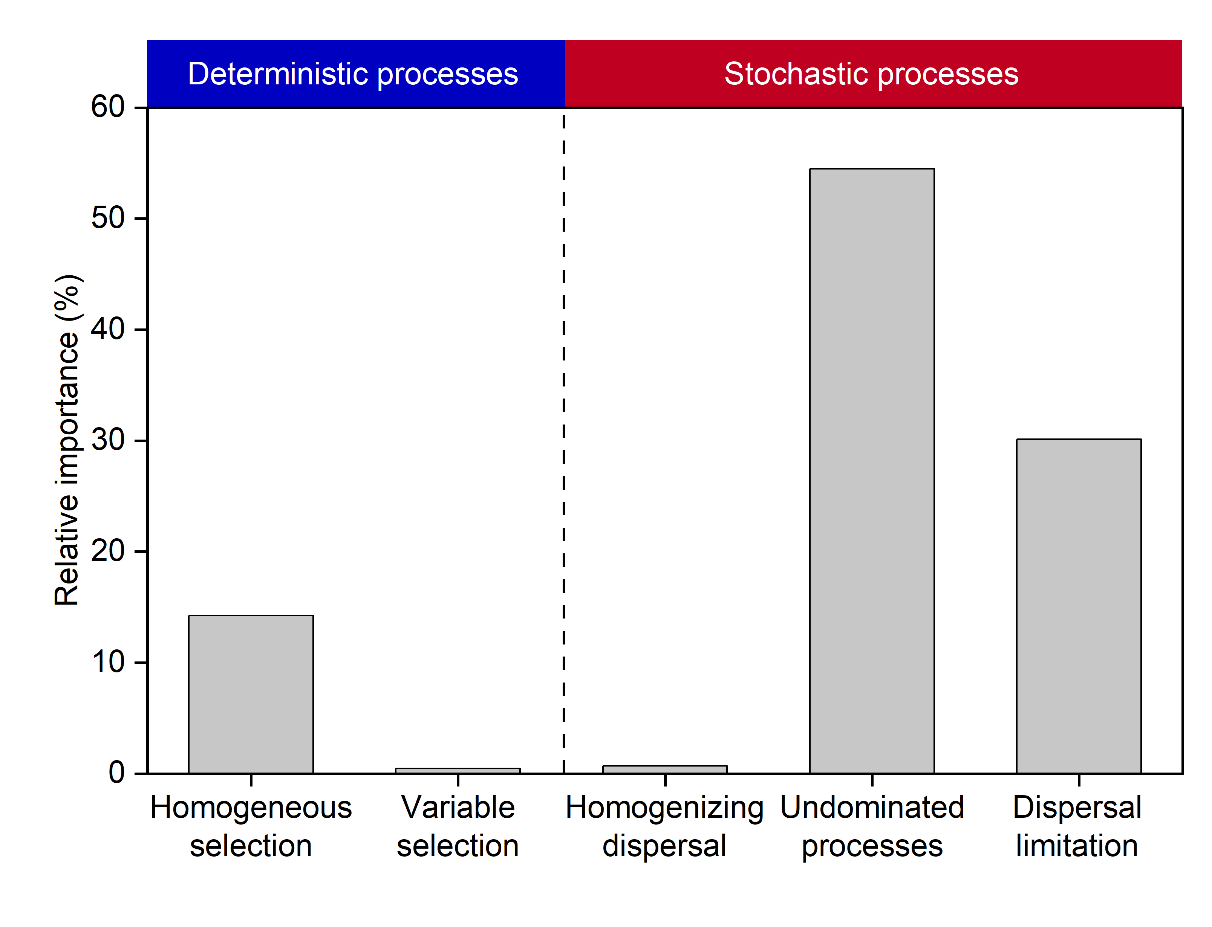


**Supplementary Figure S3.** Assembly processes of rhizosphere fungal communities in the mid-channel bar. The assembly processes were identified based on two indices of β-nearest taxon index and raup-crick index. Variable and homogeneous selection belong to deterministic processes; Dispersal limitation, homogenizing dispersal, and undominated processes are affiliated to stochastic processes.


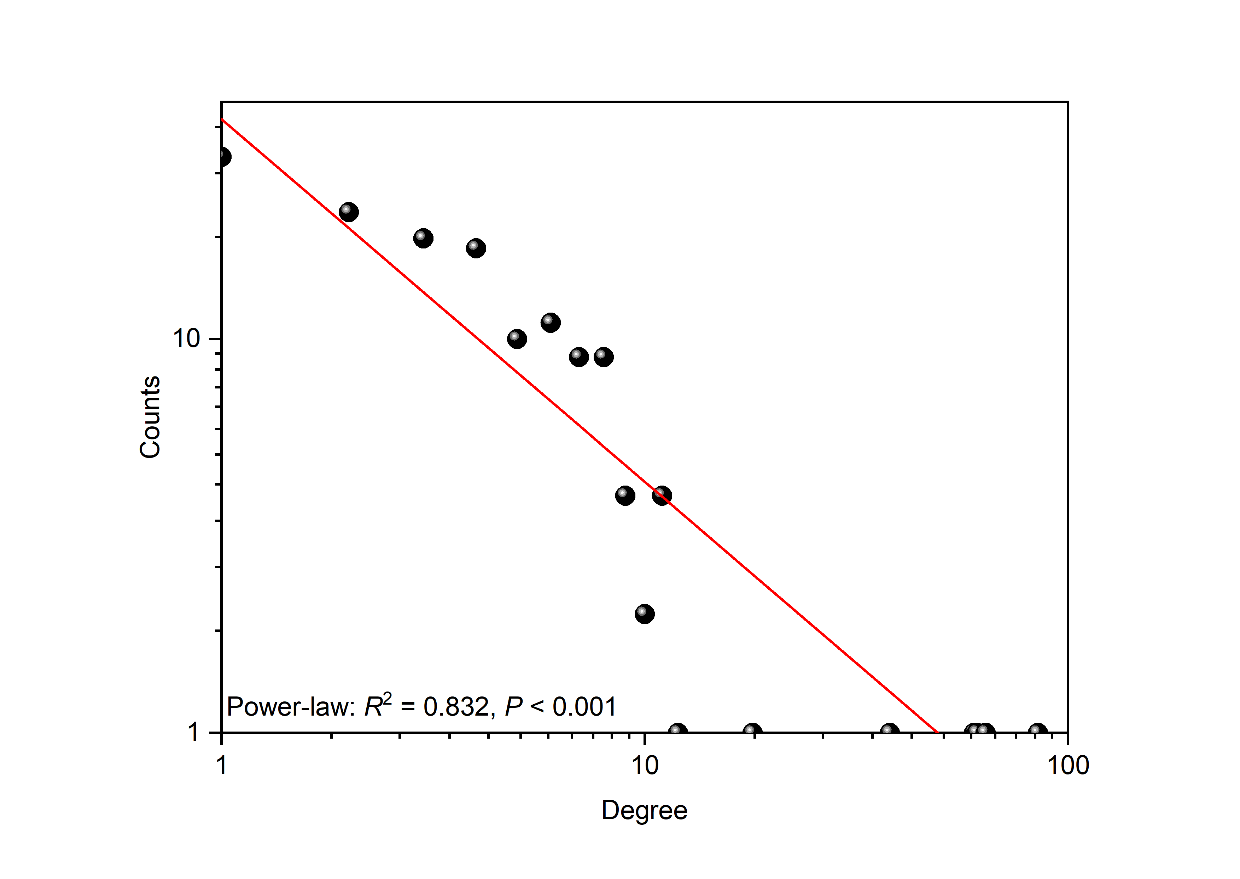


**Supplementary Figure S4.** The distributions of node degree for the real co-occurrence network of rhizosphere fungal community.
